# A model structure for describing uncertainties in benefit-risk assessment of oncology drug applications

**DOI:** 10.3389/fmed.2025.1589578

**Published:** 2025-10-15

**Authors:** Nikolaos Zafiropoulos, Francesco Pignatti, Andreas Kouroumalis, Lorenzo Guizzaro, Dominik Karres, Pierre Demolis, Olli Tenhunen, Rosanne Janssens, Anne Taams, Lourens T. Bloem, Franz Koenig, Martin Posch

**Affiliations:** ^1^Medical University Vienna, Vienna, Austria; ^2^European Medicines Agency, Amsterdam, Netherlands; ^3^Agence Nationale de Sécurité du Médicament et des produits de santé (ANSM), Paris, France; ^4^Finnish Medicines Agency (FIMEA), Helsinki, Finland; ^5^Oulu University Hospital, Oulu, Finland; ^6^Katholieke Universiteit Leuven, Leuven, Belgium; ^7^Division of Pharmacoepidemiology and Clinical Pharmacology, Utrecht Institute for Pharmaceutical Sciences, Utrecht University, Utrecht, Netherlands; ^8^Dutch Medicines Evaluation Board, Utrecht, Netherlands

**Keywords:** benefit risk assessment, uncerainty, oncology medicines, European Medicines Agency (EMA), regulatory decision making

## Abstract

**Introduction:**

The European Medicines Agency (EMA) uses structured reports to communicate the scientific review of drug applications. A European Public Assessment Report (EPAR) is published at the end of the review detailing the scientific assessment, which includes benefit-risk analysis and uncertainties in favorable and unfavorable effects. Currently, there is no detailed guidance on how to communicate uncertainties in the EPAR. This study aimed to identify uncertainties described in the benefit-risk section of a series of EPARs and derive a possible model structure for communicating them.

**Methods:**

A series of approved oncology drug applications that had used the latest EPAR template at the time of analysis was selected. The model structure was adapted iteratively, aiming to identify a small number of key elements, with input from the study team. Finally, the model structure was discussed with two experienced clinical assessors to determine clarity and potential usefulness.

**Results:**

From 64 oncology EPARs published between 2011 and 2017 (26 related to products with orphan designation), 263 uncertainties in the benefit-risk assessment were identified. The final model structure included Cause (what causes the uncertainty), Aspect (what is the uncertainty about, further described as a high-level domain and a specific component), Type (what is the kind of uncertainty, like not enough information or conflicting information), and Strategy (how the uncertainty is addressed). This four-element structure, Cause, Aspect, Type, Strategy (CATS), was discussed with expert assessors and was found to be generally understandable and relevant.

**Discussion:**

The CATS model structure has been derived as a starting point for communicating uncertainties in benefit-risk assessment of drug applications. To increase relevance, it was derived based on issues raised during actual reviews and discussed with expert reviewers. Limitations include the narrow focus of the current series and the need for validation. If found useful, this structure could eventually be used to further enhance assessment report templates.

**Conclusion:**

The proposed CATS model structure may facilitate communicating uncertainties in the benefit-risk assessment of drug applications. Further refinements, depending on the purpose and validation, aiming at broader applicability, are needed.

## Introduction

The European Medicines Agency (EMA) uses assessment reports to document and communicate the scientific assessment of drug applications for marketing authorization, conducted by leading review teams. These reports are written based on the assessment of the drug application submitted by the sponsoring company and follow detailed templates and guidelines for reporting the evaluation of the quality, non-clinical, and clinical components, including the benefit-risk assessment. The reports are shared with EMA scientific committees and the applicant during the review process, and a final version is published after after the evaluation is completed. The final report, known as the European Public Assessment Report (EPAR), is published for every application that has been approved or refused a marketing authorization and provides detailed information about the scientific assessment, including the favorable and unfavorable effects, the balance of benefits and risks, and any uncertainties. An EPAR provides the public with information on a medicine, including how it was assessed by the EMA. The EPAR is referred to in Article 13 (3) of Regulation (EC) No 726/2004, which requires EMA to publish an in assessment report for each centrally authorized medicine together with a public-friendly overview.

The EMA has been developing and refining the structure of the templates over time, adapting to changes in pharmaceutical legislation, regulatory practices, and stakeholder needs. A major revision occurred in 2011, when the EMA introduced structured templates specifically describing the benefit-risk assessment. This revision occurred as the result of a larger project led by Professor L. Phillips from the London School of Economics, aiming to optimize benefit-risk assessment and communication (2, 3). The new benefit-risk template structure followed the principles of the PrOACT-URL framework (1). This framework is an established approach to decision-making that helps to clarify and analyze complex choices. In the EMA implementation, “Pr” stands for Problem (e.g., what is the medicinal product; what is the claimed indication); “O” signifies Objectives, which involve the goals that the decision aims to achieve (e.g., identify if the benefits of the experimental treatment outweigh the risks, or if the experimental treatment is preferred to placebo) and criteria or effects (i.e., benefits and risks) for measuring achievement of such goals; “A” refers to Alternatives (the different options that can be considered, such as experimental treatment vs. placebo); “C” stands for Consequences, encouraging the evaluation of the potential outcomes and impacts of each alternative, e.g., on favorable and unfavorable effects; and “T” denotes Trade-offs between favorable and unfavorable effects. The “-URL” part of the PrOACT-URL framework refers to “U” for uncertainty (recognizing the unknown factors that can influence the consequences and therefore the benefit-risk assessment), “R” for Risk tolerance (the effect of risk attitudes when there are uncertainties), and “L” for Linked decisions (e.g., consistency with similar past decisions) (4).

In the benefit-risk section of the EPAR template, uncertainties constitute distinct subsections under the description of favorable and unfavorable effects (5). Uncertainties are also mentioned in the harmonized submission guidance for applicant companies (6). However, the templates and guidance do not provide formal definitions of what constitutes uncertainties and how to describe them. Instead, EMA reviewers are instructed to describe key uncertainties and limitations associated with the evidence supporting the proposed or final indication. The guidance recommends that uncertainties described should directly impact the benefit-risk balance and may include factors such as imprecision in effect estimates, statistical uncertainty, limitations in study design or conduct, questions regarding internal or external validity, inconsistent findings, and lack of supporting evidence from secondary endpoints. Reviewers are also invited to specify any identified information gaps and outline post-authorization measures aimed at addressing residual uncertainties or concerns. The section is meant to be updated throughout the assessment process to reflect only ongoing or unresolved issues related to quality, non-clinical, and clinical safety concerns at the respective review phase. Uncertainties that have already been resolved or are no longer relevant to the current review stage should not be described.

Without clear instructions on how to communicate uncertainties, there is a risk that reports will have varying levels of detail and information. For example, uncertainty expressed as “overall survival is uncertain” may leave readers wondering about the reason, the extent of the uncertainty, and how the uncertainty has been managed or is planned to be addressed by further studies.

The lack of guidance to describe uncertainties prompted the present analysis, aiming to provide a model structure to facilitate communication of uncertainties in the drug-regulatory setting. Using the iterative analysis of EPARs, we derived a four-element model structure (Cause-Aspect-Type-Strategy), which is proposed as a starting point for further development.

## Methods

The cohort selected for analysis of uncertainties included all EPARs published for initial applications of oncology products that received a positive opinion since the implementation of the new Committee for Human Medicinal Products (CHMP) assessment report template for benefit-risk assessment introduced in 2011 and published at the time of data cutoff (December 2017). Applications for generic, “hybrid,” and biosimilar products were excluded, as the benefit-risk assessment section usually followed a different structure. The selected cohort of EPARs was characterized in terms of orphan designation status of the respective initial application (yes vs. no), type of approval (regular vs. conditional approval or approval under exceptional circumstances), type of cancer for the approved indication (solid tumors vs. hematologic), and the main clinical study design (randomized controlled trial, RCT: yes vs. no).

The basis for identifying uncertainties was the text in the Benefit-Risk section of the EPAR under the headings “Uncertainties about Favorable Effects” and “Uncertainties about Unfavorable Effects.” Based on that text, an interpretive analysis was conducted to identify uncertainties by searching for distinct issues, following reviewers’ intended meanings and levels of detail. Uncertainties were first categorized at a high level according to the most closely related criterion for approval (efficacy, safety, and positive benefit-risk), adding categories, if necessary, as the analysis progressed. Our judgment-based, inductive approach focused on aligning with reviewers’ conceptualizations of uncertainty, without imposing predefined categories at the start. This iterative process continued until all relevant text segments could be classified into the smallest set of distinct elements. Each element was then further categorized into discrete levels or subcategories, mainly to characterize the element in a reproducible way.

The classification into respective elements was conducted independently and then as a collective exercise in case of conflicting classification (NZ, FP, LG, AK). As soon as new elements were identified or changed, new descriptions were formulated and agreed on before reclassifying all uncertainties based on the new definitions until no further changes were necessary and no ambiguities or misclassifications were apparent.

The final model structure was discussed with two expert reviewers (PD and OT) experienced in the evaluation of the benefit-risk balance for oncology products to provide initial feedback on the clarity and completeness of the model structure.

Finally, the distribution of uncertainties and associations between uncertainties and application characteristics were analyzed descriptively.

## Results

### Study cohort of oncology drugs and EPARs

In total, 64 oncology drug applications with EPARs published from January 2011 to June 2017 were included in this analysis. The characteristics of the included oncology drug applications are described in Table 1. The majority of drug applications had not received orphan designation covering the applied indication (59%), had regular approval as the outcome (72%), were indicated for the treatment of solid tumors (70%), and had an RCT as the main clinical study (73%).

**Table 1:** Marketing authorization application characteristics for the series of EPARs considered, by orphan medicinal product designation (*N* = 64).

Application	No orphan designation (*n* = 38)	Orphan designation (*n* = 26)	Total (*n* = 64)
Approval type			
Conditional/Exceptional	6 (16%)	12 (46%)	18 (28%)
Regular	32 (84%)	14 (54%)	46 (72%)
Tumor type			
Hematological	3 (8%)	16 (62%)	19 (30%)
Solid	35 (92%)	10 (38%)	45 (70%)
Main trial design			
No RCT	8 (21%)	9 (35%)	17 (27%)
RCT	30 (79%)	17 (65%)	47 (73%)

RCT, randomized controlled trial.

### Devising the model structure

The text in the sections on the benefit-risk of the EPAR described a range of uncertainties in the assessment of the evidence (sometimes explicitly referred to as “uncertainty” or “limitation”). The uncertainties were variably described in terms of deficiencies (e.g., a randomized comparative trial is lacking), the consequences (e.g., efficacy is unknown), or how to manage the uncertainty (e.g., further data are needed), without a consistent structure. Some issues were related to unexpected findings, while others were related to development choices. For some issues, remedial actions were stated (e.g., warnings and further studies); for others, no clear actions could be identified. There were variable levels of detail for each issue, from times more general (e.g., demonstration of efficacy) to more specific (e.g., safety in hepatic-impaired patients).

The textual analysis initially suggested three key elements for the model structure, which were identified by the study team as the Issue (what the uncertainty is about), the Cause (what causes the uncertainty), and the coping Strategy (how the uncertainty is managed for the benefit-risk to be positive). The most problematic category in this structure was “Issue,” as it was ambiguously interpreted in three ways: (1) the domain of the benefit-risk assessment was affected (e.g., clinical efficacy), (2) the specific component involved (e.g., “the statistical significance of the primary efficacy analysis is difficult to interpret”), or (3) the type of doubt that emerged in the mind of the assessor (e.g., “there are doubts about reliability of the analysis”). As a result, the “Issue” was split into third elements: Aspect, reflecting (1) the domain affected or (2) the precise component within the domain, and Type, reflecting the kind of doubt (final model structure).

Table 2 presents the final model structure with examples. The final four-element model structure, Cause-Aspect-Type-Strategy (“CATS”), was defined as follows:

(1) Cause (C): This element refers to the immediate data-level objective reason that is the origin of the uncertainty. For example, the lack of a randomized trial might be the Cause associated with uncertainty about efficacy.
  In our final model structure, Cause was further categorized into three main groups: Emerging Issues (e.g., unexpected results or signals), Development and Design (e.g., inappropriate study design), and Operational/Other causes (e.g., logistical issues). The main aim of these three groups was to identify issues that could be prevented, at least in theory, through more careful development strategies and regulatory guidance. Admittedly, different classifications of Cause could be defined based on different assessments and depending on the purpose.
  Although often one main immediate Cause could be found for one uncertainty, in principle, one or more Causes might be associated with one or more uncertainties. Similarly, although the chain of causes leading to an uncertainty may be endless, it is most relevant to focus on the closest one.
(2) Aspect (A): This element could be referred to as uncertain knowledge. Importantly, before classifying Aspect, the uncertainties described were translated into their *clinical* consequences, reflecting the aims of the study that focused on the benefit-risk assessment and approval of the oncology drug in question. For example, non-clinical issues about carcinogenicity would be translated into clinical safety issues. Admittedly, different choices could be made, depending on the objective.
  In this series, Aspect was generally referred to at different levels of detail, namely (a) the high-level *Domain* in the benefit-risk assessment that is affected by the issue (e.g., Efficacy) or (b) the specific *Component* of the *Domain* affected (e.g., statistical significance of the primary efficacy endpoint). Thus, we categorized *Domain* to refer to high-level domains of the regulatory benefit-risk assessment, which in the EMA template is closely related to the elements of the PrOACT-URL framework, namely, Efficacy, Safety, Benefit-Risk balance, and Other aspects (any other aspects, e.g., dosing).
  The *Component* was generally used to describe specific characteristics of the *Domain* being affected, which we labeled as Summary measure (e.g., quantitative aspects, effect size, statistical significance), Subpopulation (e.g., very few data in older patients), long-term effect (e.g., median overall survival (OS) not reached because progression-free survival (PFS) was the primary endpoint), Generalizability (external validity, e.g., issues about the efficacy in clinical practice), and Relative effect (how the drug compares to other available treatments/options). Three more Aspect *Component* categories were generally attributed to the Benefit-Risk balance *Domain* or Other aspects, namely, dose optimization, biomarker development, and drug interactions. Admittedly, different classifications of *Component*, from more to less detailed, could have been chosen, depending on the purpose.
(3) Type (T): The Type element was defined as the kind of subjective impression that arose from the Cause, such as a feeling of insufficient information (e.g., caused by lack of data), unreliable information (e.g., caused by biased adjudication of progression in an open-label study), conflicting information (e.g., discordant data in different trials), or an impression of lacking understanding of how to interpret the information (e.g., use of a “non-validated” biomarker of unknown clinical importance).
  The Type was the most debated element in the development of the final model, due to its subjective nature and the challenges of distinguishing it from the Cause. The Cause-Type dichotomy requires distinguishing between the raw, unprocessed facts as the Cause (e.g., incomplete data) and the processed, organized facts as the Type (e.g., insufficient information due to incomplete data). Furthermore, the Type was rarely described explicitly and often correlated with the Cause. However, it was considered that Causes and Types of uncertainties should be distinguished, as one Cause could lead to different Types and Strategies. For instance, the same Cause, “incomplete data” (e.g., due to a trial that was too small), may result in a Type of uncertainty of “insufficient information” (small trial in ultra-rare population) or “unreliable information” (e.g., trial stopped early for efficacy after unplanned interim analysis), which may justify different coping Strategies (e.g., warnings that effects are poorly estimated and based on strong assumptions vs. requiring additional evidence due to type I error concerns).
(4) Strategy (S): This element refers to the measures deemed necessary to ensure efficacy and safety are established, and that a positive benefit-risk assessment can be concluded. For example, a warning in the drug product information, an additional risk minimization measure, or assumption-based reasoning could serve as a Strategy. Strategies were categorized into measures aiming to reduce or acknowledge uncertainty, the latter being further categorized into six subcategories. Assumption-based reasoning was initially debated among the study team as to whether it should be classified as a distinct (uncertainty-reducing) coping Strategy rather than being just a reflection of informal evidence emerging from the data. Assumptions were finally included as Strategy when clearly expressed as statements of belief without complete or definitive evidence.

**Table 2:** Final model structure for describing uncertainties in benefit-risk assessment.

Element	Element	Description/examples
C: Cause	1. Emerging Issues	Causes are mainly due to emerging issues or unexpected results and signals, incomplete data, inaccurate data, inconsistent data, conflicting results, and measurement errors (e.g., missing data points and errors in collection).
	2. Development and Design	Causes, at least in theory, are preventable through adequate development, such as inadequate study design, small sample sizes, selection bias, lack of randomization, inappropriate controls, confounding variables, inadequate follow-up, regulatory/ethical issues, poor dose finding, and insufficient non-clinical studies.
	3. Operational/Other	Mainly external causes, such as logistical issues, resource limitations, disease too rare, environmental influences, changes in therapeutic context, and IDMC decision.
A: Aspect	A. Domain Efficacy Safety Benefit-risk balance Other aspects B. Component Summary measure Subpopulation Long term Generalizability Relative effect Dose optimization Biomarker Drug interactions	Summary measure: quantitative issues about effect size or type of the effect, mainly relating to endpoints, statistical significance, with relevance to the main trial population (e.g., unclear statistical significance of effect); issues with the type of adverse drug reaction; incidence; severity; duration; with relevance to the main population (e.g., small safety database). Subpopulation: Effects in a part of the patient population (e.g., very few data in older patients). Long-term effects: Effects of the drug in the long run, issues about sustained efficacy/safety outside the timeframe of the trial (e.g., median OS not reached because PFS was the primary endpoint), and safety issues relating to longer exposure than the trial period. Generalizability: Issues regarding the representativeness of the intended population, affecting the generalizability of the results outside the trial, i.e., external validity (e.g., issues about the efficacy in clinical practice or effectiveness). Relative effect: How the effects of the drug compare to other available treatments/options, role/value in clinical practice (e.g., outdated/missing active comparator). Dose optimization: issues with the selected dose affecting the benefit-risk profile (e.g., sparse data on a lower dose suggest similar efficacy with better safety) Biomarker: issues relevant to the development or use of a predictive or prognostic biomarker (e.g., inconclusive results from biomarker analysis) Drug interactions: issues relevant to the interaction with other products/treatments that can lead to over- or under-exposure (e.g., no data on the interaction with contraceptives).
T: Type	1. Not enough information	Insufficient data, missing data, and limited data (e.g., in the elderly).
	2. Unreliable information	Data cannot be trusted due to, e.g., quality issues or poor trial conduct, concerns about bias.
	3. Conflicting information	Divergent signals create uncertainty and a lack of consistency across studies or subgroups.
	4. Lack of understanding of information relevance	Doubt about the significance of effects, e.g., novel endpoints, rare effects, and lack of familiarity with the condition.
S: Strategy	1. Reduce the uncertainty	Systematic efforts to resolve uncertainty, e.g., further analyses, upcoming studies, planned analyses (e.g., ongoing trials), and assumption-based reasoning.
	2. Acknowledge the uncertainty	Acceptance of remaining uncertainty; measures such as warnings, routine monitoring, or assessment at the individual level.

Multiple different coping Strategies were often needed for one uncertainty. For instance, Strategies might consist of specific warnings and requesting further data.

### Examples and expert feedback

Table 3 provides two hypothetical examples structured according to the past approach and the new Cause-Aspect-Type-Strategy structure.

**Table 3:** Hypothetical examples of more conventional brief statements and the proposed comprehensive approach based on the Cause-Aspect-Type-Strategy structure.

Example	Description of uncertain knowledge	Explanation
Example 1. Demonstration of efficacy is based on a single-arm trial with an informal historical comparison of overall survival from the literature and a supportive trial in a related indication.		
Brief Statement	The submission is based on a single-arm trial, and there is uncertainty about overall survival.	Uncertainty is broadly expressed in the context of a non-randomized trial.
Proposed Comprehensive Approach	Due to a lack of randomized trials [Cause 1], it is not possible to estimate the hazard ratio for overall survival [Aspect-Component] to establish efficacy [Aspect-Domain]. The estimation presented based on published historical data [Cause 2] is not reliable [Type]. While efficacy can be assumed based on the supportive trial [Strategy 1], a further randomized trial has been requested [Strategy 2]. The lack of comprehensive evidence of efficacy has been described in the Summary of Product Characteristics [Strategy 3].	Cause: Lack of RCT and indirect comparison Aspect: Efficacy and OS Type: Unreliable information Strategy: Assumption, warning, and submit data
Example 2. Demonstration of efficacy is based on a single-arm trial with a response rate. The surrogacy of response rate for overall survival has not been established.		
Brief statement	Although treatment was associated with a high response rate, there is uncertainty about overall survival.	Uncertainty is described generally in the context of oncology development based on a biomarker with an unknown relationship to survival.
Proposed comprehensive approach	Due to the lack of demonstration of surrogacy for overall survival for the chosen biomarker response rate [Cause 1] and the lack of a randomized controlled trial [Cause 2], the claimed benefit on overall survival [Aspect-Component] is not established [Type]; however, efficacy is assumed given the high response rate [Strategy 1], and further data from a randomized trial in a related indication will be submitted to confirm the benefit in due course [Strategy 2].	Cause: Lack of RCT; surrogacy not established Aspect: Efficacy, OS Type: Not enough information Strategy: Assumption; request data

When discussed with the two clinical reviewers and among the study team, the model structure was found generally understandable and relevant, at least as a starting point. The potential for improving clarity of communication and allowing qualitative analyses was considered of interest. The need for considering the purpose of any further sub-classification of each element and a broader set of applications and assessments across different therapeutic areas, outcomes, procedures, and timepoints was discussed. The feasibility and usefulness of a systematic application of this four-element structure were discussed, given time and space constraints, for example, in the case of overarching manufacturing issues related to the active substance being unknown.

#### Illustration of the applicability of the model structure to describe the cohort

**Figure 1:**
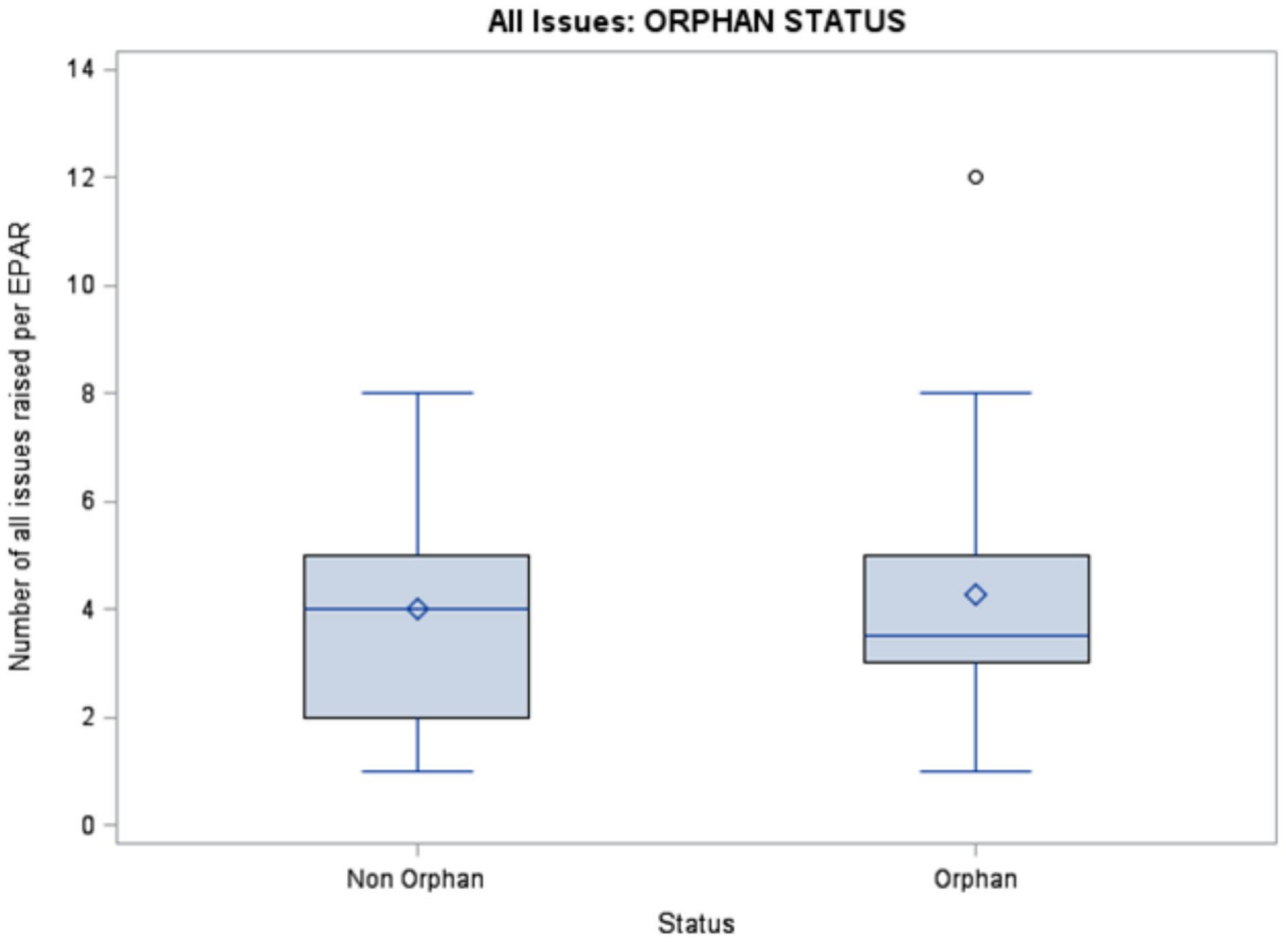
Box plot of the number of issues (uncertainties) per product for non-orphan and orphan medicinal products.

**Figure 2:**
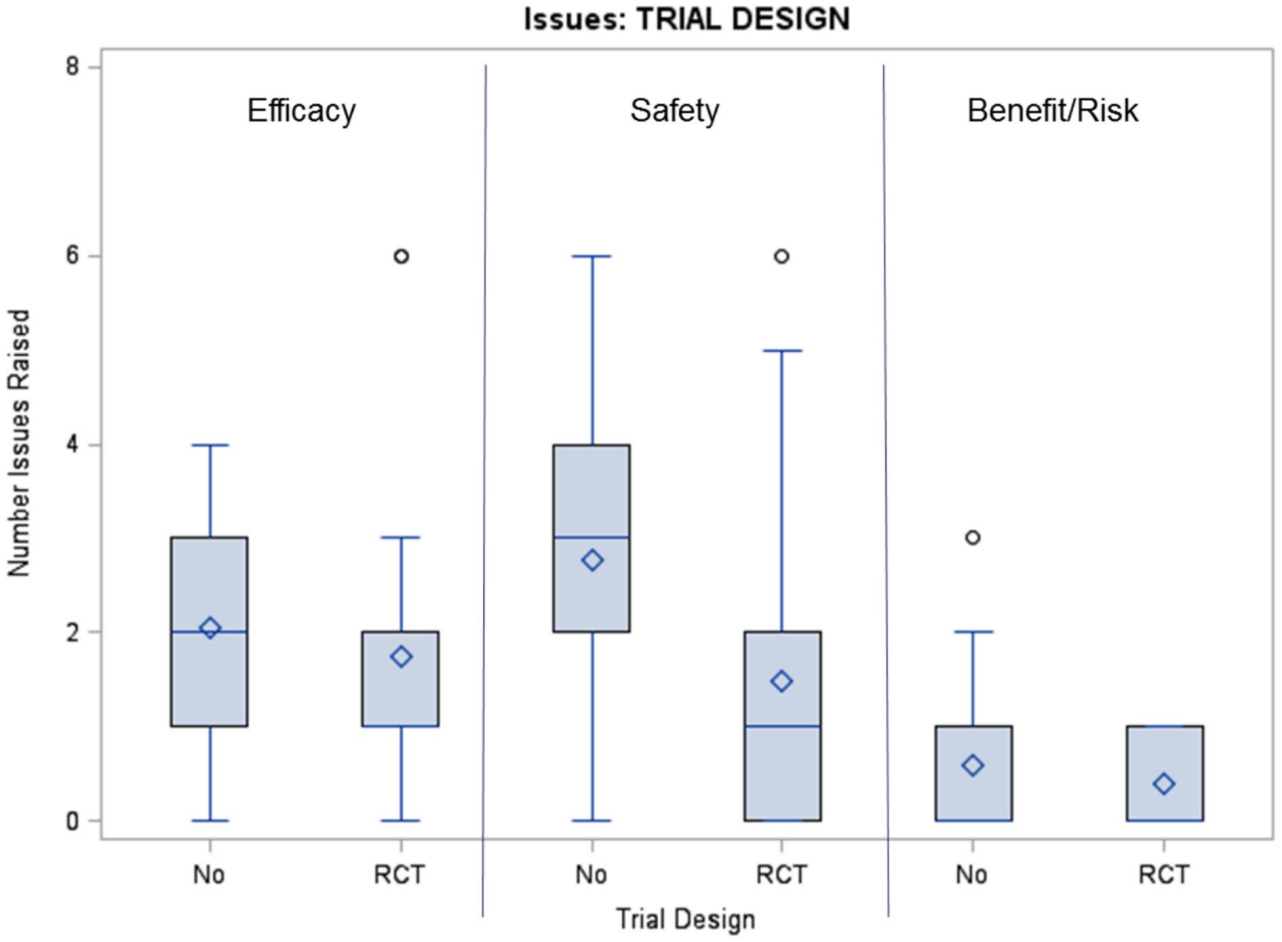
Box plot presents the number of efficacy, safety, and benefit-risk issues (uncertainties) per product by presence of a randomized controlled trial (RCT) as the main clinical study.

Descriptive statistics are presented in Figures 1, 2. The distribution of issues classified by the Cause-Aspect-Type-Strategy model structure is described in Table 4. The majority of Causes (65%) were due to development and design choices (i.e., in theory preventable). The most common aspects (Domain) were Efficacy (44%) and Safety (44%). The most common Type was “not enough information” (75%). Orphan-designated products had a higher proportion of “unreliable information” than non-orphan products (28% vs. 13%, respectively). The most prevalent Strategy was “reduce uncertainty” (67%).

**Table 4:** Distribution of uncertainties by the Cause-Aspect-Type-Strategy model, by orphan status of the marketing authorization application.

Element	Sub-element/category	No orphan designation (*n* = 152)	Orphan designation (*n* = 111)	Total (*N* = 263)
Cause	1. Emerging issues	48 (32%)	36 (32%)	84 (32%)
	2. Development and Design	97 (64%)	73 (66%)	170 (65%)
	3. Operational/Other	7 (5%)	2 (2%)	9 (3%)
Aspect-Domain	Efficacy/Benefit	66 (43%)	51 (46%)	117 (44%)
	Safety/Risk	67 (44%)	50 (45%)	117 (44%)
	Benefit–risk balance	19 (13%)	10 (9%)	29 (11%)
Type	Conflicting information	4 (3%)	6 (5%)	10 (4%)
	Lack of understanding of information relevance	2 (1%)	3 (3%)	5 (2%)
	Not enough information	126 (83%)	71 (64%)	197 (75%)
	Unreliable information	20 (13%)	31 (28%)	51 (19%)
Strategy	Acknowledge the uncertainty	51 (34%)	36 (32%)	87 (33%)
	Reduce the uncertainty	101 (66%)	75 (68%)	176 (67%)

For Cause and Strategy, only the main cause and strategy are reported for any single issue.

Orphan status drug applications had a similar number of uncertainties compared to non-orphan status drug applications (Figure 1). Products lacking randomized controlled trials were associated with a higher number of uncertainties, especially clinical safety issues (Figure 2).

## Discussion

Despite its frequent use in the regulatory setting, the term “uncertainty” remains a relatively vague concept without a formal definition, even within the scientific assessment of oncology drugs—using recent benefit-risk assessment templates—found that the concept of uncertainty encompasses a wide range of issues and findings related to deviations from the desired or required *knowledge*. Thus, rather than focusing on *uncertainty* as a concept in itself, it may be more relevant to focus on what *knowledge* is uncertain in terms of establishing the key regulatory criteria for efficacy, safety, and positive benefit-risk balance.

Following detailed analysis, the study identified the Cause-Aspect-Type-Strategy model structure to best describe uncertainties in benefit-risk assessment. The central elements of the new model structure were Aspect, which described specifically the knowledge *Domain* and *Component* that is uncertain, and Type, which referred to the feeling of doubt associated with uncertain knowledge.

The different levels of detail for presenting the Aspect, which we named *Domain* and *Component*, reflect that uncertain knowledge may present itself at different levels, from overarching uncertainties (e.g., Efficacy) to specific ones (e.g., statistical precision of an estimate of efficacy in a secondary analysis of a subgroup). This makes a quantitative classification of uncertainties challenging due to the variable levels of detail. Furthermore, the same *Component* (e.g., survival) may apply to different *Domains* (e.g., Efficacy or Safety), necessitating the use of both *Domain* and *Component* to ensure clarity.

Notwithstanding this limitation, the lack of major differences between orphan and non-orphan medicinal products in terms of the number of uncertainties was in line with previous research that has recognized that orphan status is not a predictor of a more challenging clinical development (7), potentially because uncertainties are accepted, e.g., after weighing of unmet medical need. Lack of randomized trial was associated with a higher number of safety uncertainties, compared to efficacy or benefit-risk, which may be expected on the basis of the many safety endpoints affected by the non-comparative design of the studies. Further analysis is warranted to show the association between the four different elements of uncertainty across different applications and development plans.

When it comes to communication, reviewers should strive to be as detailed as possible in terms of the Aspect, pointing out not only the *Domain* but also the *Component*, i.e., the specific knowledge and evidence in question, instead of vague, abstract concepts or generalizations that make the critique less tangible and more difficult to refute without precise details.

Type was often implicit in our series and most often debated as a separate element within the study team. Orphan-designated product applications were more often associated with unreliable information Type, which can be expected given the typical smaller study sizes and often heterogeneous characteristics. Type was a key element in another study from the decision-analysis literature showing that decision makers distinguished between three types of uncertainty: inadequate understanding, incomplete information, and undifferentiated alternatives (8). It will be important to assess if being explicit about this somewhat subjective element will be feasible in practice.

The other two elements, Cause and Strategy, were, in a sense, external to the uncertain knowledge itself and reflected the origin or mechanism that generated the uncertainty and how the uncertainty was managed, respectively. While strictly speaking, it is not necessary to describe uncertain knowledge, without a Strategy, one is left wondering if and how certain uncertainties may be acceptable. Concerning the Cause, this may help understand the other elements.

Although the study attempted to categorize the four elements further, this was admittedly an arbitrary and incomplete exercise, aiming to describe the elements themselves as they appeared in the series considered. Although these categories can help with the definition of each element, any other set of categorizations, including ones to a different level of detail, is possible and should be driven by the objectives of such an exercise.

The main strength of this study is that the proposed CATS model structure was derived from uncertainties actually described in the scientific evaluation of marketing authorization applications submitted to the EMA. This approach is grounded in practical regulatory experience, and the iterative development of the model structure, incorporating expert feedback, enhances its credibility. The application of this four-element structure may facilitate the systematic communication of uncertain knowledge that might otherwise be left implicit. This may not only improve transparency toward other stakeholders but also inform clinical and other decisions that are based on the regulatory assessment of drug applications. Additionally, the model offers a framework for tracking the evolving understanding of knowledge throughout development and post-authorization.

A limitation of this study was its narrow scope, which was based on a relatively small series of approved oncology products. Uncertainties presented for unapproved products, or those from other therapeutic areas, were not included. Generalizability to other therapeutic groups needs to be studied. Another limitation is that the model structure does not include the “seriousness” or “impact” of each uncertainty and thus its more specific relevance for the benefit-risk assessment, although this can be to some extent derived by the burden of the coping strategy (for example, a mention of the uncertainty in the SmPC vs. request of a new trial may reflect relatively less vs. more serious uncertainties, respectively).

Furthermore, the final model was reviewed by only two regulatory experts, which is not enough to establish that this structure is suitable for regulatory purposes. Formal validation would also require stakeholder input into the usefulness of such structured communication. The absence of inter-rater reliability testing or structured consensus-building methods (e.g., the Delphi technique) for classifying uncertainties also introduces potential subjectivity in applying the CATS model structure. While these limitations are acknowledged, the goal of this study was to develop a starting point to allow such further refinements or the development of different structures depending on the objectives. Thus, the CATS model structure, especially the categorizations of each of these elements, should be taken more as a starting point rather than a complete system. For instance, another study focused in more detail on different aspect domains (uncertainty categories) and aspect components (uncertainty subcategories) of uncertainty (9).

In terms of next steps, gathering feedback from a larger set of reviewers and validating its usefulness with stakeholders and readers of the reports, including industry, academia, and the general public, should be a priority. In oncology, considerations such as rare diseases, uncertainties related to expedited approvals based on non-comprehensive evidence, and real-world evidence may deserve special attention. Eventually, if deemed useful, apart from enlarging the scope of applications (outside oncology, early assessment reports, negative applications, new indications for already approved drugs, and other healthcare decision-makers), the structure with appropriate guidance could be integrated into EMA assessment report templates for assessors.

## Conclusion

This study suggests the CATS model structure as a starting point for communicating key uncertainties in the benefit-risk assessment more systematically. This structure could facilitate a complete description of uncertainties to facilitate benefit-risk assessment communication from drug-regulatory procedures toward other stakeholders. However, before potential implementation into routine assessments, further studies are needed using a broader scope of applications and gathering feedback from a broader group of assessors and readers of the EPARs.

## Data Availability

The original contributions presented in the study are included in the article/supplementary material, further inquiries can be directed to the corresponding author.
